# Reactive Organic Suspensions Comprising ZnO, TiO_2_, and Zeolite Nanosized Adsorbents: Evaluation of Decontamination Efficiency on Soman and Sulfur Mustard

**DOI:** 10.3390/toxics9120334

**Published:** 2021-12-03

**Authors:** Raluca Elena Ginghina, Adriana Elena Bratu, Gabriela Toader, Andreea Elena Moldovan, Tudor Viorel Tiganescu, Ramona Elena Oncioiu, Panaghia Deliu, Razvan Petre, Gabriel Epure, Munizer Purica

**Affiliations:** 1 Research and Innovation Center for CBRN Defense and Ecology, 225 Oltenitei Ave., 041327 Bucharest, Romania; ginghinaraluca@gmail.com (R.E.G.); adriana.bratu@nbce.ro (A.E.B.); ramona.oncioiu@nbce.ro (R.E.O.); panaghia.deliu@nbce.ro (P.D.); razvan.petre@nbce.ro (R.P.); gabriel.epure@nbce.ro (G.E.); 2 Faculty of Weapon Systems Engineering and Mechatronics, Military Technical Academy “Ferdinand I”, 39-49 George Cosbuc Boulevard, 050141 Bucharest, Romania; tiganescu.viorel.t@gmail.com; 3 National Institute for R&D in Microtechnologies, 126A Erou Iancu Nicolae Street, 077190 Voluntari, Romania; munizer.purica@imt.ro

**Keywords:** chemical warfare agents, decontamination solution, nanoparticles, GC-MS, degradation, conversion rate, soman, sulfur mustard

## Abstract

This paper comprises an extensive study on the evaluation of decontamination efficiency of three types of reactive organic suspensions (based on nanosized adsorbents) on two real chemical warfare agents: soman (GD) and sulfur mustard (HD). Three types of nanoparticles (ZnO, TiO_2_, and zeolite) were employed in the decontamination formulations, for enhancing the degradation of the toxic agents. The efficacy of each decontamination solution was investigated by means of GC-MS analysis, considering the initial concentration of toxic agent and the residual toxic concentration, measured at different time intervals, until the completion of the decontamination process. The conversion of the two chemical warfare agents (HD and GD) into their decontamination products was also monitored for 24 h.

## 1. Introduction

**Figure 1:**
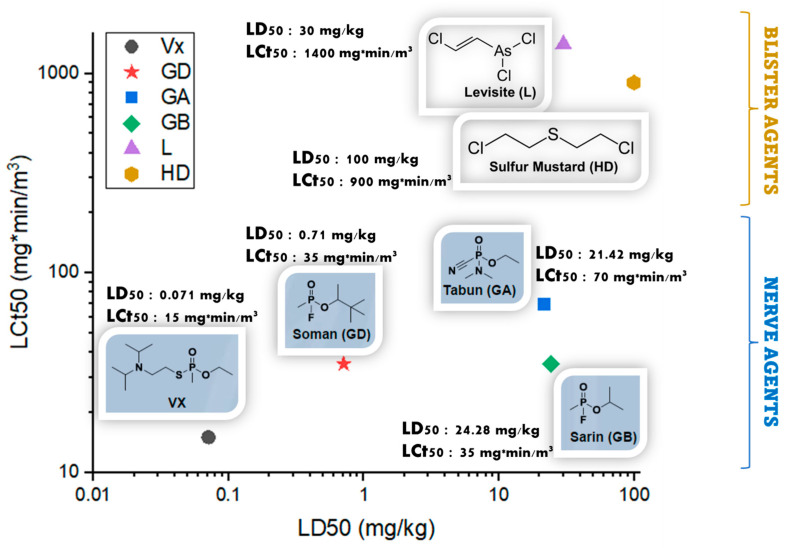
Relative toxicity of chemical warfare agents; LCt_50_ refers to lethal concentration, in mg·min/m^3^ and LD_50_ stands for median lethal dose, in mg/kg (skin exposure).

Chemical warfare agents (CWAs) represent, undoubtedly, some of the cruelest weapons of mass destruction (WMDs) created by mankind. The modern use of chemical weapons began with The First World War [1]. On 29 April 1997, the Chemical Weapons Convention [2] entered into force and officially banned the use of chemical agents. Nevertheless, large stockpiles of chemical weapons still exist. Moreover, chemical terrorism still represents an imminent threat because chemical agents are inexpensive and are relatively easy to obtain [3], even by small terrorist groups, and can cause mass casualties with small amounts of toxic. The main current CWA threats involve easily produced agents potentially manufacturable on large scales: blister agents (e.g., sulfur mustard) and nerve agents (e.g., soman, sarin, tabun or Vx). Blister agents alkylate molecules such as nucleic acids, proteins, and cellular membrane components [2]. This results in a surge of medical problems: the eyes are firstly impacted, exhibiting redness and irritation which can progress to corneal damage with photophobia, blepharospasm, and temporary blindness [2]. When in contact with skin, these highly electrophilic compounds cause the formation of large and extremely painful blisters [4]. Figure 1 illustrates a comparative representation of the toxicity of six of the most notorious CWAs, and the values plotted were selected from ref. [5].

Nerve agents are much more toxic than blister agents, because they are lethal in very small amounts: V-agents are considered the most toxic, with LD_50_ = 0.071 mg/kg (VX), followed by G-agents, LD_50_ = 0.71 mg/kg (soman) or LD_50_ = 24.28 mg/kg (sarin) [2,6]. Their mechanism of action consists of the irreversible inhibition of the acetylcholinesterase enzyme (AChE), with fatal cardiac and respiratory effects [4].

Over time, considerable efforts have been made to create efficient tools suitable for the neutralization of CWAs. The impacts of CWAs can be diminished through the neutralization of their toxic effects by employing adequate media with essential physico-chemical properties, specially designed for this purpose. The process used for the neutralization and removal of CWAs is known as ‘decontamination’. Chemical decontamination converts the toxic CW agents into less toxic and non-toxic products, which can be handled safely. The chemical reactions that are generally employed in chemical decontamination methods are either nucleophilic reactions or oxidations [7,8,9,10]. To restrict the spread of the contaminants, the decontamination must be carried out rapidly and efficiently because a CWA event represents an emergency which can result in injury, illness, or loss of life. Therefore, emergency intervention departments/offices need to have the appropriate facilities, equipment, and capabilities to respond to chemical agents’ exposure, in order to save lives and to mitigate injuries. In this context, the decontamination of warfare agents has become a major challenge for researchers as well as for soldiers. The efficiency of decontamination is conditioned by a set of variables: contamination time, nature of the agent and nature of decontaminants, type of surface contaminated, contamination density, and decontamination formulation, pH, and temperature [3]. An ideal decontamination formulation should be highly compatible with the chemical agents to be able to perform a rapid and efficient decontamination while being non-corrosive and stable in storage. Even though several decontamination formulations have been developed so far, there are intrinsic limitations of their application: low solubility of the CW agents, low neutralization efficiency, toxicity, and corrosivity [11]. Bleaching powder, calcium hypochlorite, and sodium hypochlorite were the first decontaminants that were employed for CWA neutralization. Many commercially available decontamination formulations have been developed over time. Solutions based on ethanol, phenol, sodium hydroxide, ammonia, calcium hypochlorite, tetrachloroethylene, surfactants, and water were developed and were introduced in the endowment of the armies, but they presented drawbacks related to their poor performances at certain pH levels and also to the potential damages produced to the decontaminated substrate (metal [12], glass [13], plastics [14], rubber [15], wood flooring [16], concrete [17], painted surfaces [18], or the surface of sensitive equipment [19], etc.). Solid adsorbents, such as Fuller’s earth [20], have been used as an alternative because they do not affect the surface subjected to decontamination, but the major inconvenience in this case is that they only physically remove the contaminants, not neutralize them [11]. Active sorbents, also called destructive sorbents, represent a prospective development in this field. These solid materials absorb CWAs on their surface, followed by chemical decomposition. Unfortunately, practical CWAs decontamination studies have shown that not all solid decontamination sorbents react with CWAs to the required degree of decomposition [21].

In contrast, nanosized particles (NPs) have been reported as promising prospective reactive sorbent materials [7,11,22] which have the ability to potentiate the neutralization of CWAs [23,24]. In comparison with the conventional materials, nanostructures have attracted great interest due to their enhanced reactivity towards CWAs, owing to large specific surface and reactive edges, corner defects and unusual lattice planes [22]. Prasad et al. reported the decontamination of sulfur mustard (HD) and its surrogate, 2-chloroethyl ethyl sulfide (CEES) by manganese oxide nanosheets and nanotubes and also by titania nanotubes [11,22,25]. CWAs underwent degradation on the surface of these nanostructures through hydrolysis reactions [4,11,22]. Crystalline porous oxides known as zeolites [26] can also adsorb and potentially detoxify simulants and CWAs [27]. When employed in the appropriate decontamination formulations, the nanoparticles can improve the efficiency of CWA decontamination.

Regarding the formulations employed for chemical decontamination, they can be either aqueous or organic, or even biphasic. Certain studies have demonstrated that aqueous systems may delay the oxidation process of CEES simulants while an organic solvent would bring a benefic contribution to the oxidation reaction [28]. Moreover, mustard agents and their analogous simulants are highly soluble in organic solvents. In some cases, for the inactivation of nerve agents, such as soman, a 10% NaOH aqueous solution may prove to be efficient, but with a consumption norm of about 0.5–1 L/m^2^, specific for aqueous decontamination solutions, making this decontamination process a producer of high amounts of liquid wastes, whereas an organic decontamination solution has a consumption norm of 0.5 to 0.1 L/m^2^. These aqueous decontamination solutions also have the inconvenience of being highly corrosive [16].

In other cases, an organic decontamination solution can be more practical because it includes some advantages, in comparison with aqueous systems: the consumption norm of decontamination solution per square meter can be considerably lower; the preferential solubility of CWAs in specific organic solvents recommend the utilization of organic solutions for the improvement of the decontamination capacity; the compatibility between the organic solvents and CWAs leads to shorter reaction times; organic formulations have better resistance to low temperatures than aqueous systems; organic system are more stable in storage and, in general, they do not require additional preparation at the contaminated site, thus improving the response time of the decontamination actions; and using lower amounts of solvents, the organic systems do not require additional operations prior to analysis (such as extraction and concentration in case of an aqueous solution).

Taking into consideration the need for extensive studies on real warfare agents and for a better understanding of the systems that streamline the decontamination process, we herein report an original survey on novel highly reactive organic suspensions comprising ZnO, TiO_2_, and zeolite nanosized adsorbents together with the evaluation of their decontamination efficiency on soman and sulfur mustard. As far as we are concerned, there are scant data in the literature about the influence of these types of nanostructures on the decontamination efficiency of real CWAs in organic media.

The novelty of this study consists of the development of new organic decontamination formulations, with enhanced decontamination efficiency brought by the nanosized particles selected for this type of application, and also in revealing, by thorough investigations, how they influence the degradation of two real warfare agents: a blister agent—sulfur mustard (HD), and a nerve agent—soman (GD).

Thus, in this article, we describe the preparation of three different types of NP-based decontamination formulations and an extensive evaluation of their decontamination efficiency for HD and GD warfare agents by means of a GC-MS technique. We also identified and quantified the main degradation products of GD and HD produced during the decontamination process.

## 2. Materials and Methods

### 2.1. Materials

The materials employed for the preparation of the decontamination formulations—2-ethoxyethanol (≥99.8%, ethylene glycol monoethyl ether, Sigma Aldrich, St. Louis, MO, USA ), monoethanolamine (≥98%, Sigma Aldrich, St. Louis, MO, USA), sodium hydroxide (≥98%, Sigma Aldrich, St. Louis, MO, USA), isopropyl alcohol (≥99.7%, Sigma Aldrich, St. Louis, MO, USA), and sodium lauryl sulfate (SDS, Sigma Aldrich, St. Louis, MO, USA)—were used as received. Nanosized adsorbents ZnO, TiO_2_, and zeolite were provided by the National Institute for Research and Development in Microtechnologies (IMT, Bucharest, Romania), they were obtained according to ref. [29] (ZnO), ref. [30] (TiO_2_), ref. [31] (zeolite-NPs) and were used as received, for the decontamination solutions.

For decontamination tests, real chemical warfare agents were utilized: bis(2-chloroethyl) sulfide (HD, sulfur mustard, purity: 95%, CAS: 505-60-2, Schedule 1A(4) in the Chemical Weapons Convention (CWC), own synthesis) and soman (GD, Pinacolyl methylfluorophosphonate, purity 90%, CAS: 96-64-0, Schedule 1A(1) in the CWC, own synthesis). The sample preparations for the GC-MS analyses involved dichloromethane (≥99.8%, DCM, Merck Millipore, Burlington, MA, USA) extractions, anhydrous sodium sulphate (Sigma Aldrich, St. Louis, MO, USA) drying and derivatization with N,O-Bis(trimethylsilyl)trifluoroacetamide (≥99%, BSTFA, Sigma Aldrich, St. Louis, MO, USA) silylation reagent. All the tests involving the CWA utilized in this study were performed at the Research and Innovation Center for CBRN Defense and Ecology, in the ‘Chemical Analysis Laboratory’, the OPCW Designated Laboratory from Romania.

### 2.2. Methods

#### 2.2.1. Synthesis of Decontamination Solutions

The organic decontamination solution (SD) was prepared in a three-neck flask equipped with dropping funnel with pressure compensation, ascending refrigerant, and mechanic stirrer. The components of the decontamination formulations were added progressively, maintaining a temperature of 30 °C with the aid of a cooling bath. Ethylene glycol monoethyl ether (50–60 wt.%) was the first reagent introduced in the three-neck flask, followed by the dropwise addition of monoethanolamine (20–30 wt.%). Meanwhile, a solution of sodium hydroxide 48 wt.% was prepared. After completing monoethanolamine addition, NaOH solution (2–5 wt.%) was introduced dropwise in the decontamination solution. In the meantime, sodium lauryl sulfate (1–3 wt.%) was dissolved in isopropyl alcohol (10–20 wt.%). Afterwards, this solution was slowly added to the decontamination formulation. The last step consisted of the dispersion of the nanosized adsorbents with the aid of a probe sonicator (750-Watt Ultrasonic Processor, 30 min at 40% amplitude). Table 1 summarizes the decontamination formulations obtained.

Therefore, the last stage of the synthesis of the reported decontamination solutions consisted of preparing four suspensions with different concentrations of NPs (0.1, 0.5, 1 and 2 wt.% NPs in SD) for each type of nanosized adsorbent (ZnO, TiO_2_, and zeolite), as detailed in Table 1.

#### 2.2.2. Preliminary Evaluation of the Neat Organic Decontamination Solution

The density of the organic decontamination solution was calculated with the aid of a pycnometer. The alkalinity of the neat organic decontamination solution was determined by titration with HCl (1N). All measurements were effectuated in triplicates and the mean values were reported.

The SD was especially designed not to damage the decontaminated surfaces and not to affect the operational capability of military equipment. In this way, the solution has been tested in accordance with AEP-7 (nuclear, biological, and chemical (NBC) defense factors in the design, testing, and acceptance of military equipment), by immersing painted metallic samples in SD for 30 min, rinsing with water, drying, and evaluating the samples immediately and after 24 h, in accordance with ISO 4628-2:2016, for cracking, exfoliation, discoloration or other visible defects. The solution was exposed to cycles of extreme temperature and humidity conditions characteristic for the Romanian region and neighborhoods (−33 °C to +49 °C), i.e., two cycles (24 h each), one for low temperatures (extreme temperature −33 °C) and one for high temperatures (extreme temperature +49 °C), provided by NATO AECTP 230 standard (climatic conditions) [32], and afterwards, it was investigated whether it preserved its decontamination efficiency.

#### 2.2.3. Decontamination Procedure

The evaluation of the decontamination efficiency of the novel organic formulations on real CWA, HD, and GD was performed in two main stages: controlled contamination with CWA followed by sample preparation of the decontaminated samples for GC-MS analyses.

Five milliliters from each of the twelve synthesized suspensions were contaminated with 5.25 µL HD, with 5.55 µL GD, meaning 1000 ppm of pure toxic in the suspension. The decontamination process was performed under magnetic stirring (300 rpm) at room temperature (20 °C). Two control samples (SD) were also contaminated with 1000 ppm toxic. After specific decontamination times (2 min, 10 min, 30 min, 60 min, 3 h, 5 h, and 24 h), 200 µL of each suspension were extracted with 3.8 mL dichloromethane (DCM), dried for water traces over sodium sulphate, filtered on 45 µm Sartorius filter, and analyzed by GC-MS/EI. In order to identify and quantify the degradation products produced after the decontamination process, 1 mL sample was derivatized with 20 µL BSTFA (N,O-Bis(trimethylsilyl)trifluoroacetamide) at 60 °C for 30 min and analyzed by GC-MS. BSTFA is a derivatizing agent widely used in the derivatization of low or no volatility compounds, thereby resulting in the formation of trimethylsilyl (TMS) derivative.

For the evaluation of the decontamination efficiency, the decontamination factor (*DF*) was calculated taking into account the initial concentration of the toxic chemical and the residual toxic after decontamination, resulted from the GC-MS investigations. The formula for the calculation of the decontamination factor is: $DF=100*\left(C_{0}-C_{f}\right)/C_{0}$, where DF is the decontamination factor, $C_{0}$ is the initial toxic concentration, and $C_{f}$ is the final concentration, indicating the residual contamination. Controlled contamination and the decontamination procedure, followed by GC-MS analyses, were repeated in triplicates and the average values obtained were reported.

### 2.3. Characterization

The morphology and dimensions of ZnO, TiO_2_, and zeolite NPs were examined by an ultra-high-resolution field emission scanning electron microscope (FE-SEM)—FEI Company Nova NanoSEM 630. The characterization was performed at magnifications of 120 kx and 100 kx, and acceleration voltages of 10 kV and 15 kV, through the lens SE detector (TLD). The FT-IR spectra of the decontamination solutions were obtained at 4 cm^−1^ resolution, from 550 to 4000 cm^−1^, with the aid of a Pekrin Elmer Spectrum Two instrument, ATR mode. Gas chromatography mass spectrometry analyses were performed on a GC Thermo Scientific Trace 1310–TSQ 9000 triple quadrupole MS. GC analysis method: carrier gas—helium, 1.5 mL constant flow, 270 °C injector temperature, splitless injection mode, TR5MS gold column, 5% phenyl 95% dimethylpolysiloxane phase, 30 m × 0.25 mm × 0.25 μm, temperature program—40 °C to 300 °C with a heating rate of 10 °C/min. MS analysis method: solvent delay—2.5 min and 8.5 min (derivatization method), electron impact (EI) ionization mode, 40 eV electron energy, 40–650 *m*/*z* scan range. Qualitative analyses and the identification of the toxic compounds and their degradation products were performed by comparing the mass spectra of the chemicals with NIST (National Institute of Standards and Technology) and OCAD (OPCW Central Analytical Database) spectra libraries. Quantitative analyses were performed with the addition of an internal standard.

## 3. Results and Discussion

The first step in our study consisted of the development of the organic decontamination solution, which subsequently served as the dispersion media for the investigated nanoparticles. The synthesis of the organic decontamination solutions, with and without the nano adsorbents, is described in the Methods section. Before adding the NPs, the neat organic decontamination solution (SD), considered as a blank sample for these decontamination experiments, was subjected to a series of specific analyses in order to obtain some preliminary information. Therefore, following the above-described procedures, we obtained decontamination solutions with 0.91 ± 0.02 g/cm^3^ density and total alkalinity of 2.4678 ± 0.5 cm^3^ HCl 1 N/1 g analyzed solution. The neat organic solution did not degrade the tested painted metallic surfaces; therefore, we can affirm that these solutions do not affect the operational capability of military techniques when employed for decontamination. In addition, this solution proved that it maintained its decontamination efficiency even after being exposed to multiple cycles of extreme temperature and humidity conditions.

The second step of this research involved the addition of three types of nano adsorbents, ZnO, TiO_2_, and zeolite, to the organic decontamination solution, in order to investigate their ability of enhancing the decontamination efficiency for HD and GD. The morphology of the nanosized adsorbents employed in the decontamination solutions was investigated through SEM analysis.

**Figure 2:**
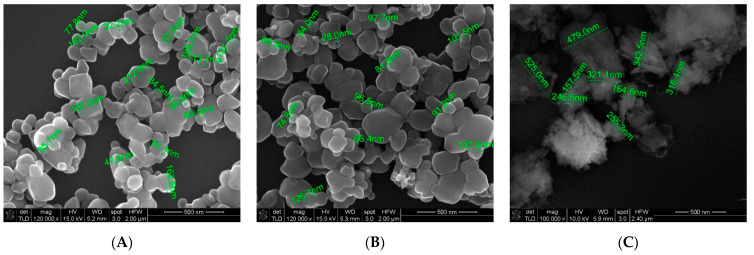
SEM images of the NPs employed in the decontamination solutions: (**A**) ZnO NPs; (**B**) TiO_2_ NPs; (**C**) zeolite NPs.

In Figure 2, it can be observed that the morphology of the ZnO (Figure 2A) and TiO_2_ (Figure 2B) nano adsorbents was very similar. Their dimensions can also be measured on comparable scales, the sizes of the particles varying between 60 and 100 nm in both cases. The zeolite adsorbent has a different morphology due to its porous structure (Figure 2C). The size of the particles measured falls within 100–500 nm, being larger than ZnO and nanoparticle-sized TiO_2_. The nanosized adsorbents presented in Figure 2 were used as received for the decontamination suspensions, by simply being added and sonicated (details in the Methods section) in the organic solution, prior to the decontamination stage.

**Figure 3:**
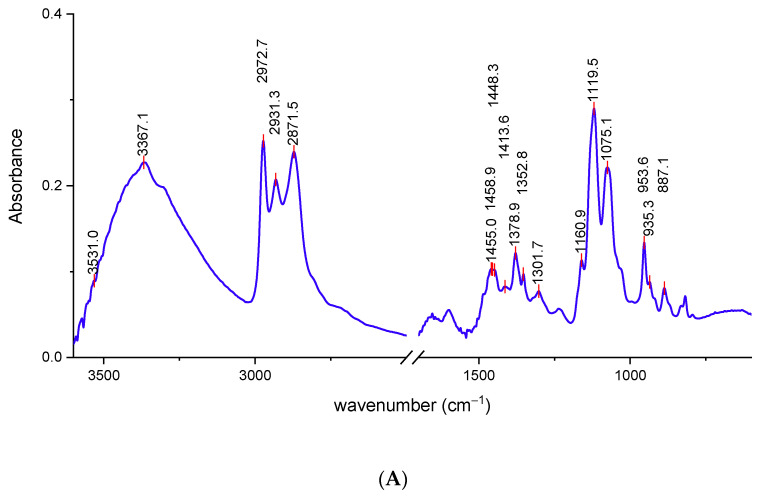

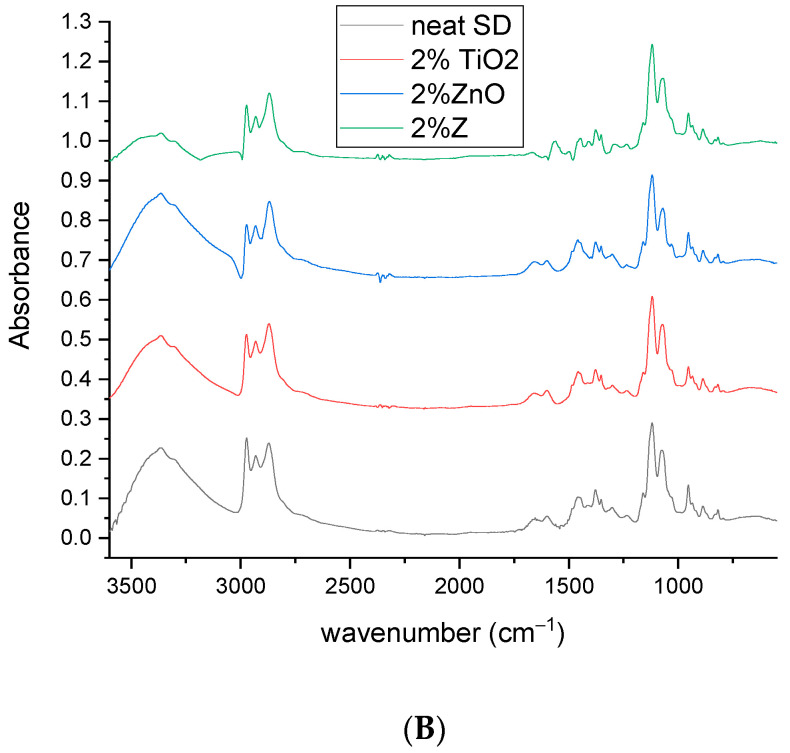
FT-IR spectra: (**A**) neat SD and (**B**) decontamination solutions with NPs.

The chemical composition of the decontamination solution and the interactions between its components and the CWAs directly influences the decontamination performance. Comparative FT-IR plots for neat SD and for the decontamination solutions enriched with different concentrations of nanosized adsorbents are illustrated in Figure 3.

The broad peak specific for O-H stretching vibrations partially overlapped with the peak specific for N-H stretching vibrations in the 3500–3300 cm^−1^ region, due to the presence of monoethanolamine in the decontamination solution (Figure 3A). A characteristic absorption band of the C–H stretching bond could be observed around 2973 cm^−1^. The high intensity of this peak can be attributed to the overlap of the absorptions of a large number of C–H bonds [33] from –CH_2_– groups present in the DS, particularly those found in the structure of the surfactant. The peaks found at 2973 cm^−1^ and 2871 cm^−1^ can be associated with C–H stretching from CH_3_ groups. Peaks situated at around 1450 cm^−1^ and 1378 cm^−1^ can also be assigned to methyl groups. The intense sharp peak found at 1119 cm^−1^ can be attributed to C–O stretching. Primary alcohols from SD composition exhibited a strong C–C–O asymmetric stretch at around 1075 cm^−1^. The decontamination solutions are complex blends; therefore, some of the adsorption bands, specific for a certain component, may possibly overlap with those of the other components, and thus some of them may have been omitted because they were not clearly visible on the spectra. Even so, the FT-IR analysis offers valuable information about the main groups present in the structure of the components from the decontamination solutions.

The decontamination tests on real warfare agents offered the possibility of evaluating the decontamination performances of the solutions developed in this study. A comprehensive assessment of the decontamination capacity of the organic solutions enriched with ZnO, TiO_2_, and zeolite nanoparticles for GD and HD was effectuated as described in Methods section, and it is detailed below.

**Figure 4:**
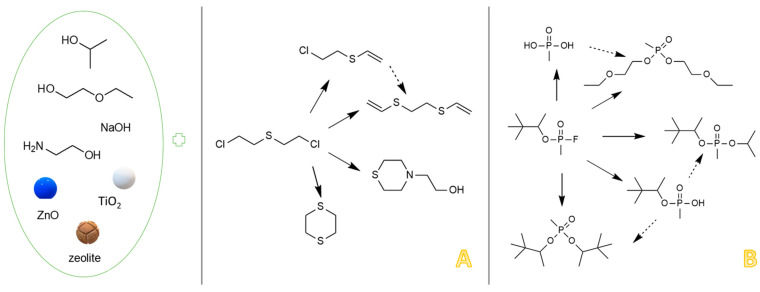
Schematic illustration of the main degradation products of HD (**A**) and GD (**B**) obtained through the decontamination process with SD-NP suspensions.

The main degradation products of CWA obtained in this study are summarized in the schematic illustration found in Figure 4.

The interaction of each type of decontamination solution with HD and GD was thoroughly investigated and the results are explained below.

**Figure 5:**
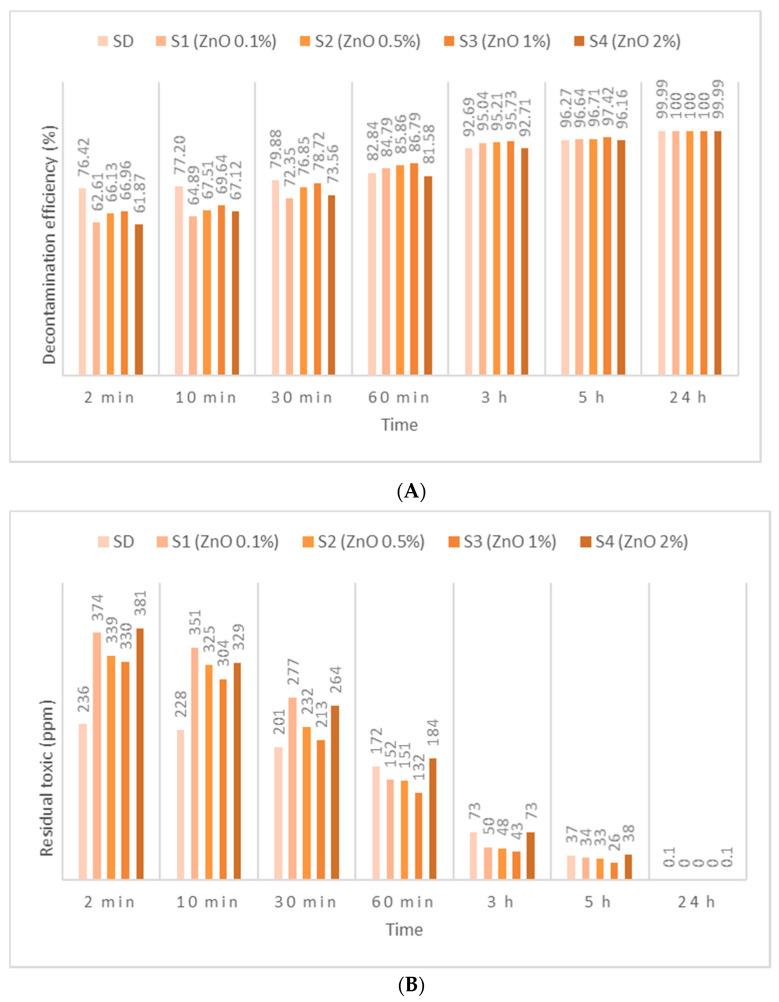
(**A**) HD decontamination efficiency with organic decontamination suspensions of ZnO NPs with concentrations of 0.1%, 0.5%, 1%, 2%, and (**B**) HD residual concentrations after the decontamination process.

**Figure 6:**
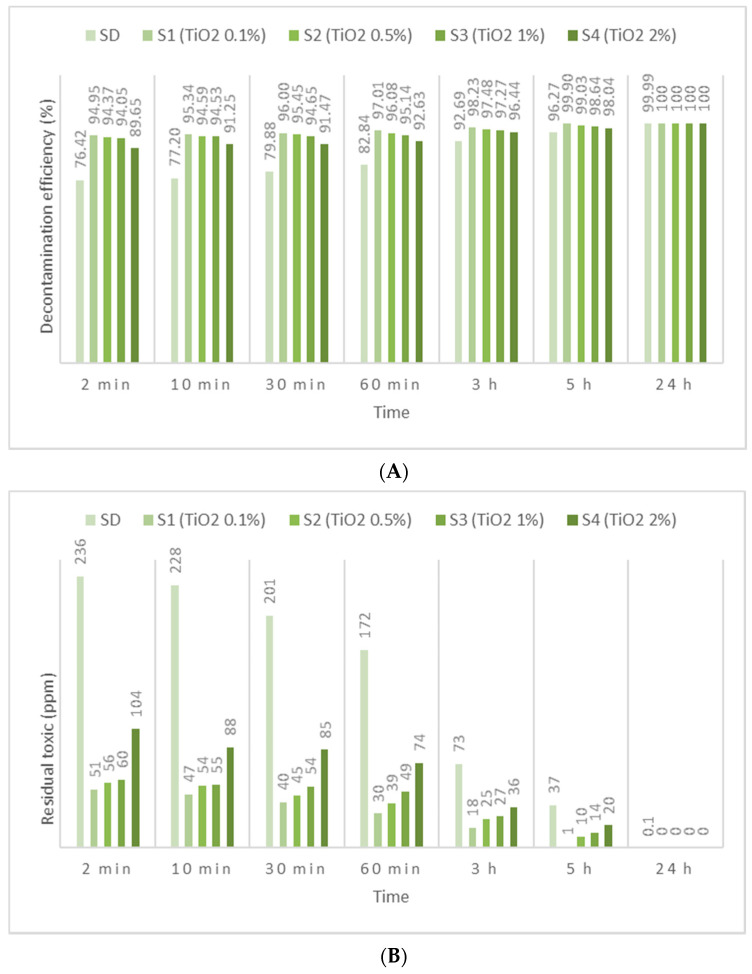
(**A**) HD decontamination efficiency with organic decontamination suspensions of TiO_2_ NPs with concentrations of 0.1%, 0.5%, 1%, 2%, and (**B**) HD residual concentrations after the decontamination process.

**Figure 7:**
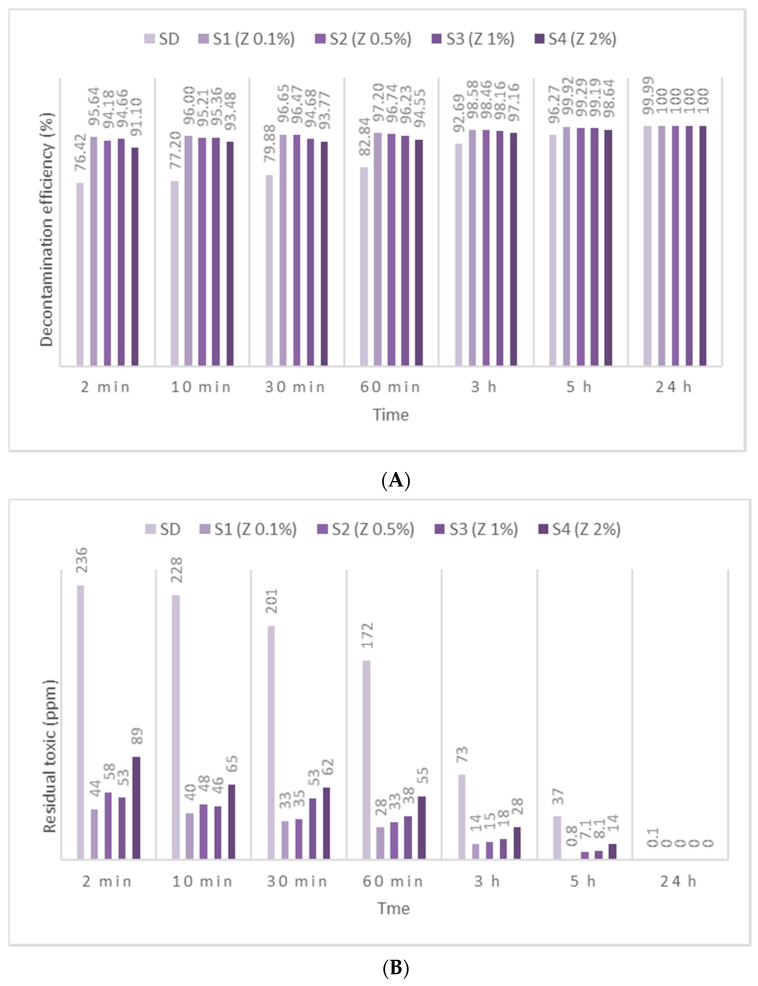
(**A**) HD decontamination efficiency with organic decontamination suspensions of zeolite NPs with concentrations of 0.1%, 0.5%, 1%, 2%, and (**B**) HD residual concentrations after the decontamination process.

HD employed in this study has been identified by *m*/*z* (109, 111, 63, 158) and by comparing the spectra with NIST and OCAD analytical libraries. HD decontamination tests performed on the neat organic solution showed a very fast decontamination reaction, leading to a decontamination degree (Figure 5A, Figure 6A and Figure 7A) of 76.42% after 2 min. The decontamination reaction progressed another 6.5% in the next 58 min and continued advancing by 3.5%/h in the next 4 h. The residual HD (Figure 5B, Figure 6B and Figure 7B) indicated concentrations decreasing from 236 ppm (after 2 min) towards 37 ppm (after 5 h), from an initial contamination of 1000 ppm. After 24 h, the decontamination process could be considered complete with a 99.99% decontamination efficiency. The next step consisted of evaluating the influence of the nanosized adsorbents on the decontamination efficacy of HD. Therefore, SD—1% ZnO NP suspensions—revealed decontamination efficiencies (Figure 5A) between 66.96% (immediately after initial contact meaning 2 min) and 97.42% (5 h), with an increase in decontamination efficiency of 19.83%/h in the first hour and slowing down to approximately 2.65%/h in the next 4 h. The residual HD after decontamination with S3—1% ZnO suspension—indicated values (Figure 5B) starting from 330 ppm (after 2 min) and reaching 26 ppm (after 5 h). The decontamination efficiency was 1.15% higher compared with one of neat SDs after 5 h. Evaluating the four concentrations of ZnO NP suspensions, we observed a higher decontamination efficiency with the increase in the concentration from 0.1% to 1%. The suspension with 2% ZnO NPs exhibited lower decontamination efficacy, probably due to the agglomeration of the NPs, which may have slowed down the reaction rate and reduced the overall active surface available for the adsorption of toxic. SD—0.1% TiO_2_ NP suspensions reacted instantly and through an almost complete decontamination reaction with HD, showing a decontamination efficiency (Figure 6A) of 94.95% (after 2 min), 97.01% (after 1 h) and 99.90% (after 5 h). After an almost instantaneous behavior of the decontamination reactions, the conversion rates continued to increase by another 2.06% in the first hour; afterwards, it started to slow down to 0.72%/h in the next 4 h. The residual HD after decontamination with S1—0.1% TiO_2_ NPs, showed values (Figure 6B) between 51 ppm (2 min) and 1 ppm (5 h). The decontamination efficiency of S1—0.1% TiO_2_ NPs after 5 h was higher with 3.63% in comparison with SD. When comparing the immediate decontamination efficiency (after 2 min) we can affirm that the decontamination efficiency of SD-TiO_2_ NPs (0.1 wt.%) was 18.53% higher than that of neat SD, decisive fact in operational field. SD-zeolite NP suspensions shower slightly superior results than SD-TiO_2_ NP suspensions. The initial decontamination reaction showed a higher conversion rate of about 95.64% (after 2 min), and even a slightly higher decontamination efficiency of 99.92%, after 5 h (Figure 7A). In the first hour, the conversion rate continued to increase by another 1.56%, and approximately 0.68%/h in the next 4 h. The residual HD (Figure 7B) firstly indicated 44 ppm (after 2 min) and 1 ppm later (after 5 h). The decontamination efficiency was 3.65% higher in comparison with that of neat SD after 5 h, and 19.22% higher after only 2 min. SD-zeolite NPs (0.1 wt.%) showed the best decontamination efficiency of HD. After 24 h, all decontamination solutions showed a decontamination efficiency of about 99.99% or 100%. Analyzing Figure 5, Figure 6 and Figure 7, we can affirm that for all TiO_2_ NPs and zeolite NP suspensions, we observed that decontamination efficiency slightly decreased with the increase in NP concentration, whereas for ZnO NP suspensions, the optimal concentration for decontamination seemed to be 1 wt.%, because these solutions exhibited the best results.

**Figure 8:**
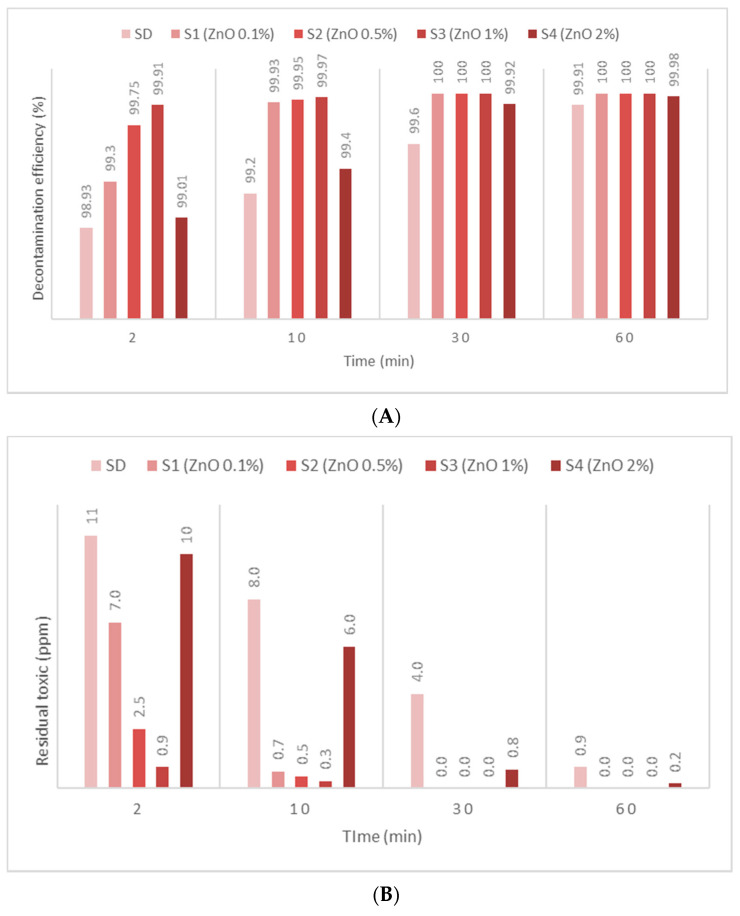
(**A**) GD decontamination efficiency with organic decontamination suspensions of ZnO NPs with concentrations of 0.1%, 0.5%, 1%, 2%, and (**B**) GD residual concentrations after the decontamination process.

**Figure 9:**
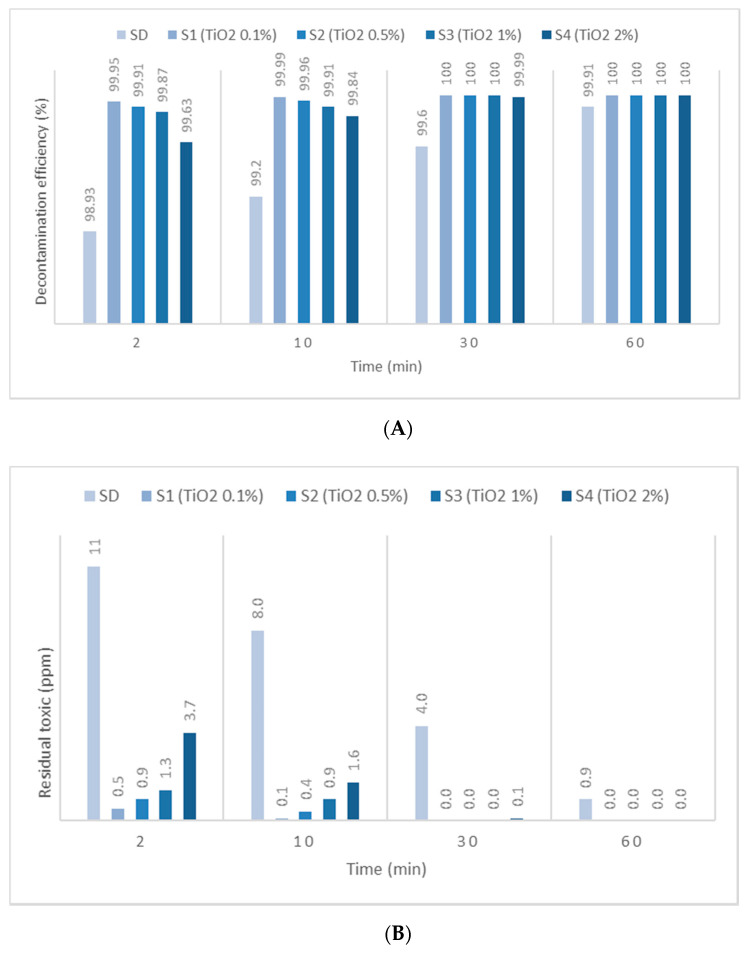
(**A**) GD decontamination efficiency with organic decontamination suspensions of TiO_2_ NPs with concentrations of 0.1%, 0.5%, 1%, 2%, and (**B**) GD residual concentrations after the decontamination process.

**Figure 10:**
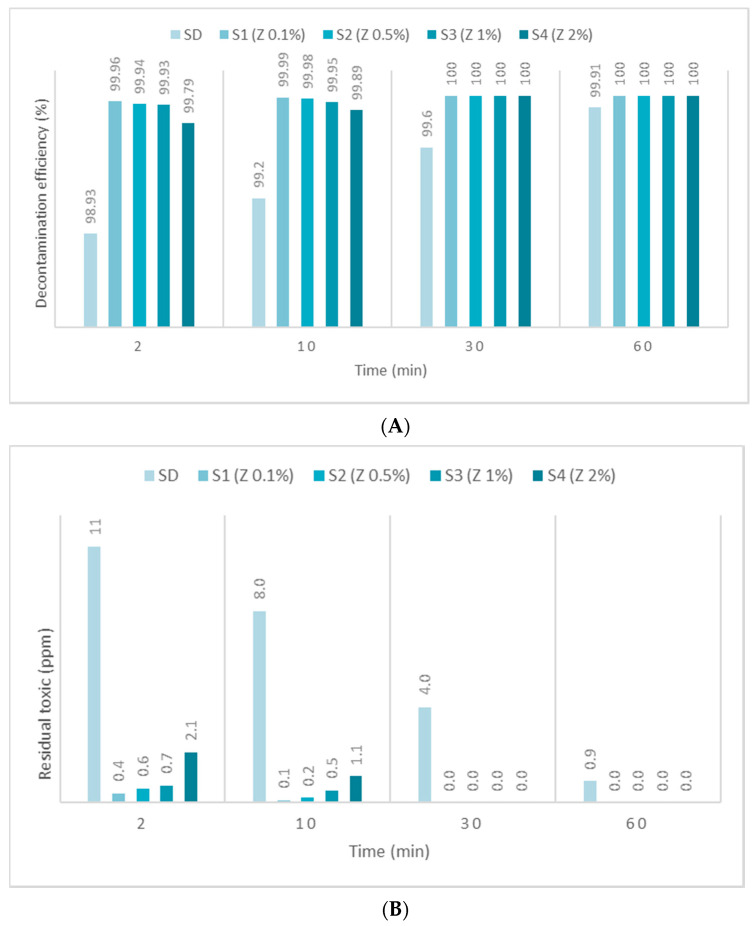
(**A**) GD decontamination efficiency with organic decontamination suspensions of zeolite NPs with concentrations of 0.1%, 0.5%, 1%, 2%, and (**B**) GD residual concentrations after the decontamination process.

The second warfare agent investigated in this study, GD, was identified by *m*/*z* (99, 126, 82, 69) and by comparing the spectra with NIST and OCAD analytical libraries. GD decontamination tests showed a very fast decontamination reaction, leading to a decontamination degree of 99.83% after 2 min. The decontamination progressed by another 0.98% in the next 58 min, leading to a decontamination efficiency of 99.91% and an equivalent of 0.9 ppm (after 1 h) from an initial contamination of 1000 ppm. SD-ZnO NP suspensions showed an instant and almost complete decontamination reaction, exhibiting a decontamination efficiency (Figure 8A) of 99.91% after 2 min and 99.97% after 10 min (for SD—1% ZnO), with equivalents of 0.9 ppm and 0.3 ppm GD remnants, respectively, after the decontamination process (Figure 8B). The decontamination efficiency was 0.98% higher compared with SD after 2 min. Comparing the four concentrations of ZnO NP suspensions, we observed a higher decontamination efficiency when increasing the concentration of ZnO from 0.1% to 1%. The suspension with 2% ZnO NPs showed poorer results, probably due to the agglomeration of the NPs, thus decreasing the adsorption surface and lowering the efficiency of decontamination. SD—0.1% TiO_2_ NP suspensions showed an instant decontamination efficiency of 99.95% and a complete reaction after 10 min (Figure 9A), equivalent of 0.5 ppm and 0.1 ppm GD remanent after decontamination (Figure 9B). SD—0.1% zeolite NP suspensions showed an instant decontamination efficiency of 99.96% and a complete reaction after 10 min (Figure 10A), equivalent of 0.4 ppm and 0.1 ppm GD remanent after decontamination (Figure 10B). In both cases, the decontamination efficiency was approximately 1% higher than neat SD after 2 min. Comparing the four concentrations for both TiO_2_ NPs and zeolite NP suspensions, we observed that decontamination efficiency decrease was directly proportional with the increasing concentration of the NPs, and that some differences appeared between 1% and 2% concentrations. SD-zeolite NPs (0.1 wt.%) showed the best decontamination efficiency of HD. The decontamination reactions involving NP suspensions were all completed after 30 min, and in the case of SD, the decontamination was finished after more than 1 h. In the case of TiO_2_, and the zeolite NP (0.1 wt.%) suspension, the reaction was completed after 10 min.

**Figure 11:**
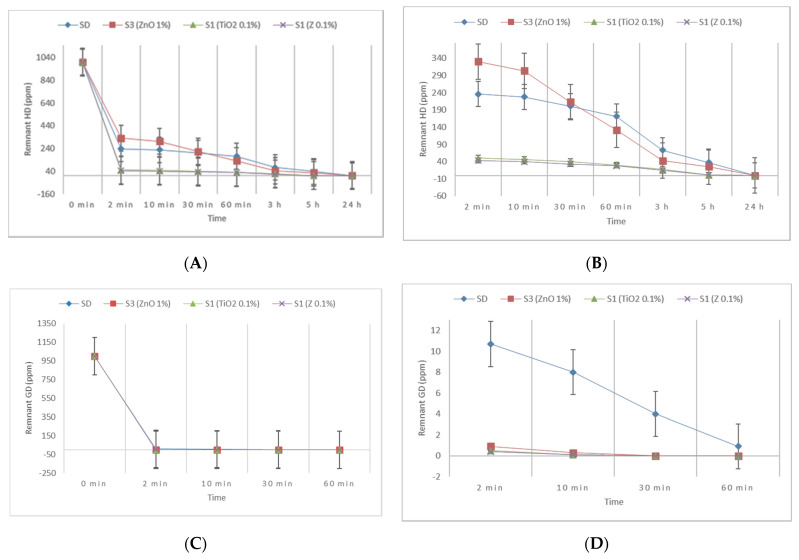
Remnant contamination with HD (**A**) and GD (**C**) after decontamination with S3-ZnO, S1-TiO_2_, S1-Z, and neat SD; (**B**) detail extracted from (**A**) for smaller ppm ranges of HD; (**D**) detail extracted from (**C**) for smaller ppm ranges of GD.

The solutions that exhibited the highest decontamination efficiency from each class of NP suspensions (S3-ZnO, S1-TiO_2_, S1-Zeolite), together with the neat organic solution (SD), were presented comparatively, considering their residual contamination for HD and for GD, as can be observed in Figure 11. It was observed that the organic suspensions containing 0.1 wt.% of TiO_2_ or zeolite reduced the decontamination time from 1 h to 10 min for GD. In the case of HD, the decontamination efficiency of these suspensions after 2 to 10 min was equivalent to the decontamination efficiency of the neat organic solution after 5 h, circumstances which may have had a crucial role in the operational field. Thus, by analyzing all the results illustrated above, we can affirm that the hypothesis about the possibility of enhancing the decontamination efficiency of our organic solution through the addition of small amounts (0.1–1 wt.%) of NPs (ZnO, TiO_2_, or zeolite) was confirmed and demonstrated through reproducible results.

The final step of this work consisted of identifying the decontamination products produced in this process, quantifying their abundance proportionally with time, evaluating the conversion rate of the CWA into its main decontamination products. Four main degradation products resulting from the decontamination of HD, and five main degradation products resulting from the decontamination of GD were identified and are illustrated in Figure 4.

**Figure 12:**
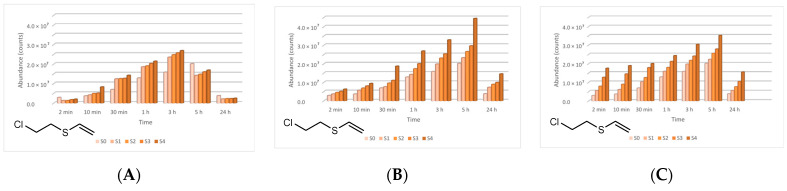
2-Chloroethyl vinyl sulfide abundance in time in the HD decontamination process with (**A**) ZnO, (**B**) TiO_2_, and (**C**) zeolite suspensions.

**Figure 13:**
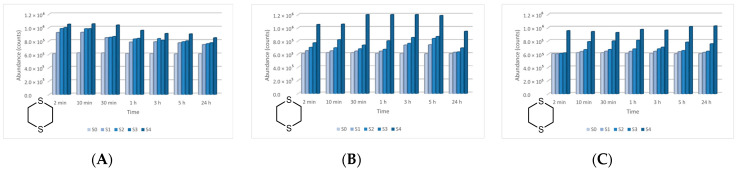
1,4-Dithiane abundance in time in the HD decontamination process with (**A**) ZnO, (**B**) TiO_2_, and (**C**) zeolite suspensions.

**Figure 14:**
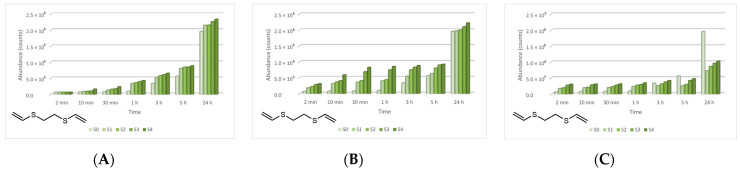
1,2-Bis(vinylthio)ethane abundance in time in the HD decontamination process with (**A**) ZnO, (**B**) TiO_2_, and (**C**) zeolite suspensions.

**Figure 15:**
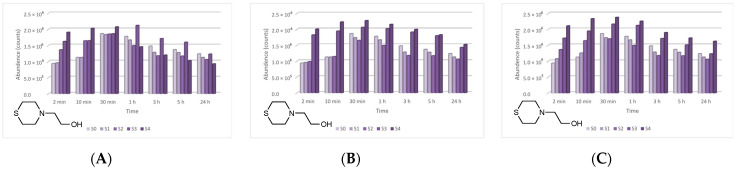
Thiomorpholinoethanol abundance in time in the HD decontamination process with (**A**) ZnO, (**B**) TiO_2_, and (**C**) zeolite suspensions.

The four main degradation products resulting from the decontamination of HD were identified and quantified by the GC-MS technique. The first degradation product, 2-chloroethyl vinyl sulfide (CAS 81142-02-1, Non-Schedule—N.S.) was identified by *m*/*z* (73, 122, 60, 45) at a retention time (RT) of 6.45 min. This product was slightly visible at the beginning of the decontamination process (after 2 min) in small concentrations, and its concentration increased to a maximum value after 3 h. After this point, the concentration dropped, showing that this compound had started to transform into a subsequent decontamination product. Its concentration after 24 h was equivalent to the concentration measured after 2 min (Figure 12). A linear concentration increment of 2-chloroethyl vinyl sulfide was observed as the concentration of nanoparticles in the suspension increased in all the three cases. The second degradation product of HD found by GC-MS investigation was 1,4-dithiane (CAS 505-29-3, N.S.), which was identified by *m*/*z* (120, 46, 61, 45) at RT for 9.11 min. The abundance of this compound in the decontamination solution was almost constant at any point of the decontamination process, which sustains the hypothesis that the compound existed at time zero of the decontamination process, and the neat decontamination solution had a neglectable effect in this case. Even so, the concentration of this compound increased in the NP suspensions, directly proportional with the NP concentrations, which demonstrates the enhanced formation of degradation products in the suspensions containing nanoparticles (Figure 13). In the specific case of SD—2 wt.% TiO_2_, the abundance of 1,4-dithiane was double than that in the case of neat SD. The third degradation product formed was 1,2-bis(vinylthio)ethane (CAS 4413-12-1, N.S.), identified by *m*/*z* (45, 59, 87, 58) at RT for 10.60 min. This degradation product showed a linear increase with time, up to 5 h from the start of the decontamination process. In the time interval between 5 h and 24 h, an exponential increase in its concentration was observed (Figure 14). A slight increment of its abundance with the increase in NP concentration has also been observed. The last main degradation product of HD identified in this study was 2-thiomorpholinoethanol (CAS 6007-64-3, N.S.). This chemical compound was identified as TMS-derivative, in the silylated samples with BSTFA, N-(2-trimethylsilyloxyethyl) thiomorpholine (CAS 959040-20-1) by *m*/*z* (116, 88, 73, 204) at RT for 14.34 min. Its formation started at the beginning of the decontamination process and increased to a maximum after 30 min; afterwards, it slowly decreased. In a particular case, SD—2 wt.% ZnO, this maximum was reached after 1 h (Figure 15). The decrease in concentration after a specific moment leads to the idea that this compound was transforming into a different decontamination product.

**Figure 16:**
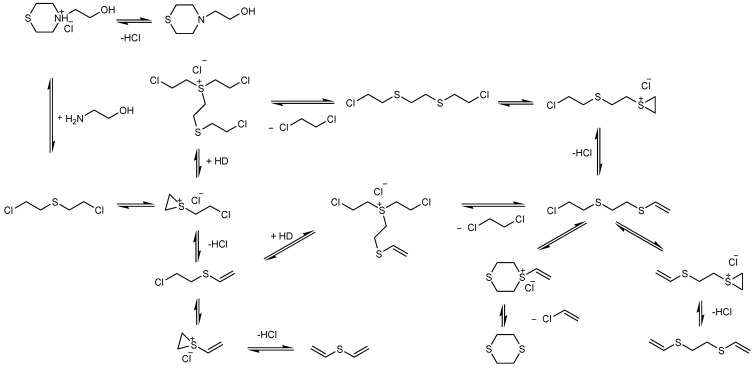
Hypothetical degradation mechanism for HD.

The proposed mechanism of reaction for the degradation of HD is schematically illustrated in Figure 16.

**Figure 17:**
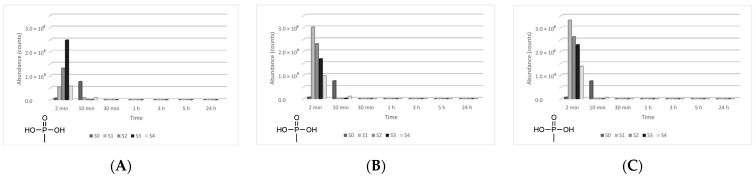
Methylphosphonic acid abundance as a function of time in the process of GD decontamination with (**A**) ZnO, (**B**) TiO_2_, and (**C**) zeolite suspensions.

**Figure 18:**
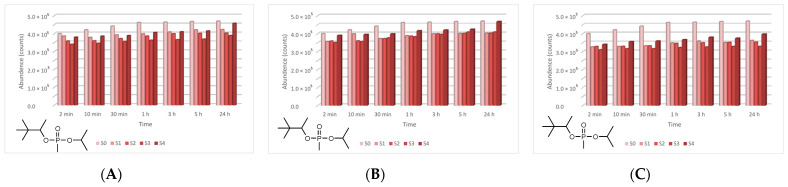
Isopropyl pinacolyl methylphosphonate abundance as a function of time in the process of GD decontamination with (**A**) ZnO, (**B**) TiO_2_, and (**C**) zeolite suspensions.

**Figure 19:**
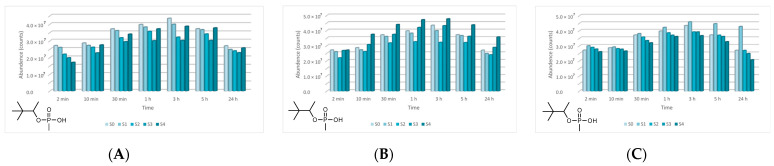
Pinacolyl methylphosphonic acid abundance as a function of time in the process of GD decontamination with (**A**) ZnO, (**B**) TiO_2_, and (**C**) zeolite suspensions.

**Figure 20:**
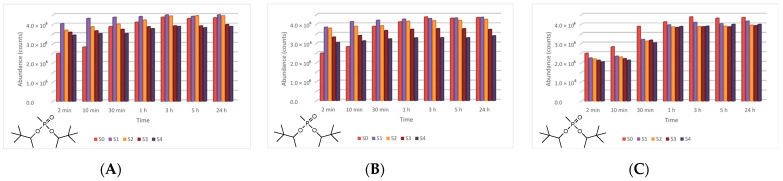
Dipinacolyl methylphosphonate abundance as a function of time in the process of GD decontamination with (**A**) ZnO, (**B**) TiO_2_, and (**C**) zeolite suspensions.

**Figure 21:**
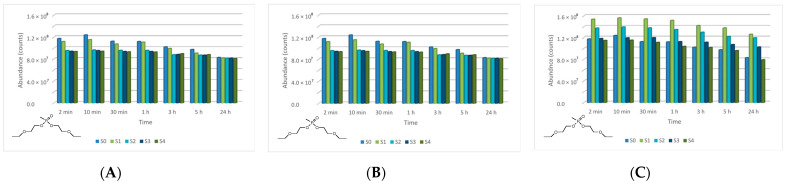
Methylphosphonic acid, di(2-ethoxyethyl) ester abundance as a function of time in the process of GD decontamination with (**A**) ZnO, (**B**) TiO_2_, and (**C**) zeolite suspensions.

In the case of GD, five major degradation products resulted from its decontamination. These compounds were identified and quantified by GC-MS technique as well. The first degradation product of GD, methylphosphonic acid (CAS 993-13-5, Schedule 2B(4)), was identified as di-TMS-derivative in the silylated samples with BSTFA, bis(trimethylsilyl) methylphosphonate (CAS 18279-83-9) by *m*/*z* (225, 73, 226, 147) at RT of 10.44 min. This compound appeared after 2 min, but after this moment its abundance dropped down to zero. In case of ZnO suspensions, the highest concentration of methylphosphonic acid, after 2 min, was observed in the case of S3 (1 wt.% ZnO) (Figure 17). The other suspensions which presented a lower concentration of this GD degradation product showed a delay in the process of conversion, indicated by the fact that the product has also been observed after 10 min in small concentrations. In both cases, TiO_2_ and zeolite, the highest concentration of this degradation product, after 2 min, was observed in the case of S1 (0.1 wt.% NPs). Delayed conversion was also observed (Figure 17), in comparison with ZnO suspensions. The second GD degradation product found was isopropyl pinacolyl methylphosphonate (CAS 92411-67-1, Schedule 2B(4)), which has been identified by *m*/*z* (123, 97, 124, 125) at RT of 12.24 min. Its formation started at the beginning of the decontamination process and continued with a slightly increasing trend (Figure 18). The decontamination product showed a higher abundance in neat SD, in comparison with NP suspensions. The third GD degradation product identified was pinacolyl methylphosphonic acid (CAS 616-52-4, Schedule 2B(4)). This chemical compound was identified as TMS-derivative in the silylated samples with BSTFA, pinacolyl trimethylsilyl methylphosphonate (CAS 199116-10-4) by *m*/*z* (153, 169, 195, 151) at RT of 12.78 min (Figure 19). Pinacolyl methylphosphonic acid was the second most abundant decontamination product of GD in this decontamination process. Its concentration reached a maximum at 3 h from the beginning of the decontamination process, which suggests the idea that it further transformed into another degradation product. The fourth degradation product of GD, dipinacolyl methylphosphonate (CAS 7040-58-6, Schedule 2B(4)) was identified by *m*/*z* (123, 97, 124, 85) at RT for 15.22–15.25 min. This compound was formed from the beginning of the decontamination process (2 min) and its abundance exhibited a slightly increasing trend (Figure 20). A more visible increment was observed in zeolite suspensions (Figure 20C). The last main degradation product of GD was methylphosphonic acid di(2-ethoxyethyl) ester (CAS 6069-07-4, Schedule 2B(4)), identified by *m*/*z* (72, 45, 123, 97) at RT for 15.35–15.50 min. It was the most abundant decontamination product, formed in high amounts from the beginning of the process and continued with a slightly decreasing trend (Figure 21). It is highlighted that the 0.1 wt.% NP suspensions showed a better conversion of GD into this chemical, compared with the other NPs solutions. As NP concentrations increased, GD showed a decreasing tendency of converting into methylphosphonic acid di(2-ethoxyethyl) ester.

**Figure 22:**
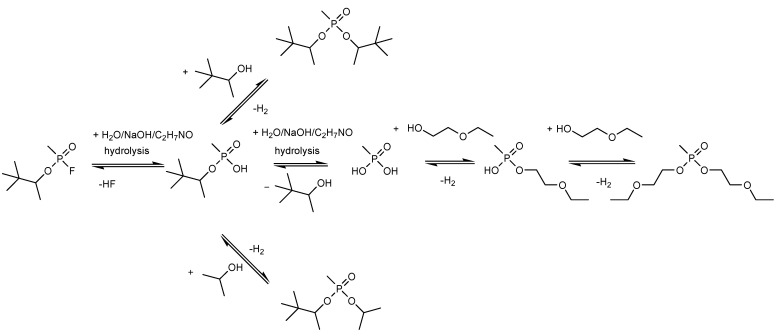
Hypothetical degradation mechanism for GD.

The proposed mechanism of reaction for the degradation of GD is schematically illustrated in Figure 22.

**Figure 23:**
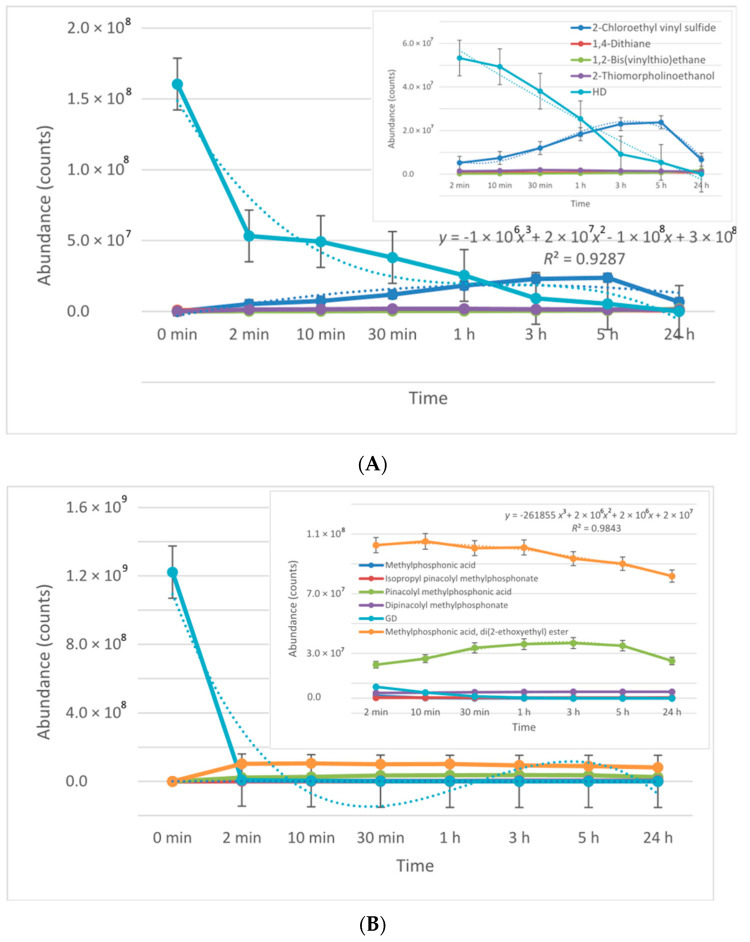
The conversion rate of the toxic chemicals (**A**) HD and (**B**) GD into their degradation products during 24 h decontamination process.

The conversion rates of the chemical warfare agents into degradation products were evaluated and presented in Figure 23A,B. In the decontamination process of HD, the major decontamination product was 2-chloroethyl vinyl sulfide. This compound was formed from the beginning of the reaction and its concentration continued to increase until 3 h had elapsed. After this moment, its concentration dropped down, which leads to the assumption that 2-chloroethyl vinyl sulfide was probably transformed into 1,2-bis(vinylthio)ethane. After 5 h, the concentration of 1,2-bis(vinylthio)ethane increased exponentially. On the other hand, 1,4-dithiane was found in small concentrations, as impurity in the HD was tested. However, in the decontamination process utilizing NP suspensions, an increase in its concentration of about 40% to 50% was observed, compared with the neat organic solution. Another specific degradation product for these organic compositions was 2-thiomorpholinoethanol, which resulted from the reaction between HD and monoethanolamine (present in the organic solution, 20–30 wt.%). In the decontamination process of GD, the most abundant and specific degradation product was methylphosphonic acid, di(2-ethoxyethyl) ester. This compound resulted from the reaction between GD and 2-ethoxyethanol (present in over 50% in the organic decontamination formulation). The reaction took place almost instantly, with a high conversion rate. Pinacolyl methylphosphonic acid is the second most abundant degradation product observed. The concentration of this chemical compound exhibited an increasing trend in the first 3 h. After this moment, the concentration started to decrease, probably because pinacolyl methylphosphonic acid tends to transform into dipinacolyl methylphosphonate and isopropyl pinacolyl methylphosphonate, both compounds displaying higher concentrations after this point. Isopropyl pinacolyl methylphosphonate resulted from the reaction between GD and isopropyl alcohol (present in 10–20 wt.% in the organic decontamination solution).

The relative acute toxicity of the obtained degradation products is included in category 3 and 4, meaning slightly toxic and irritating, non-toxic and non-irritating, respectively. The LD50 for these two categories ranges between 500 and 5000 mg/kg, respectively; over 5000 mg/kg for oral toxicity and between 2000 and 5000 mg/kg, respectively; and over 5000 mg/kg for dermal toxicity, as stated by the Environmental Protection Agency (EPA). For the degradation products methylphosphonic acid, di(2-ethoxyethyl) ester, dipinacolyl methylphosphonate, thiomorpholinoethanol, 1,2-bis(vinylthio)ethane, no toxicological data are currently available. In fact, the purpose of a decontamination process does not seek to eliminate toxicity altogether, but to transform highly toxic substances into substances that are not harmful to humans.

## 4. Conclusions

An organic decontamination solution, which subsequently served as dispersion media for three different types of nanoparticles, especially designed for the decontamination of chemical warfare agents, was synthesized and characterized. Density, pH, and total alkalinity of this organic solution were assessed, and the specific preliminary tests demonstrated that it preserved its decontamination performances after being exposed to multiple cycles of extreme temperature and humidity conditions, in accordance with NATO standard AEP 58 requirements for chemical warfare decontamination solutions [33]. The next step consisted of obtaining three types of reactive organic suspensions (based on ZnO, TiO_2_, and zeolite nanosized adsorbents), briefly characterized by means of FT-IR and SEM analyses.

In this study, the main focus was on the comprehensive examination of the decontamination performances of these solutions for two real chemical warfare agents: GD and HD. For this purpose, the initial concentration of toxic agent and the residual toxic concentrations were determined and compared with the aid of a GC-MS technique. Measuring the concentration of residual toxic agent at different time intervals offered evidence on the progression of the decontamination process. It was observed that the decontamination efficiency for HD slightly decreased with the increase in NP concentration, whereas for ZnO NP suspensions, the optimal concentration for decontamination seemed to be 1 wt.%. The positive influence of NPs on the enhancement of the decontamination performance was more noticeable in the case of soman decontamination. The decontamination organic suspensions showed higher decontamination factors for GD than the neat organic solutions. In comparison with HD, which required between 5 and 24 h for complete decontamination, the complete degradation of GD was fulfilled in a maximum of 60 min, due to the different chemical properties of these two chemical warfare agents. When comparing the improvements brought by the nanosized adsorbents, we observed that in the case of HD, TiO_2_ and zeolite led to higher decontamination factors more rapidly than ZnO, whereas for GD, they behaved in a similar manner.

The conversion of the two chemical warfare agents into their decontamination products was also monitored up to 24 h. Four main degradation products resulting from the decontamination of sulfur mustard, and five main degradation products resulting from the decontamination of GD were identified and quantified by the GC-MS technique as well.

We can conclude that these organic suspensions comprising ZnO, TiO_2_, and zeolite nanoparticles proved their decontamination efficiency on soman and sulfur mustard, whereas extensive study on their conversion into harmless degradation products offers a comprehensive image on the decontamination process.

## Figures and Tables

**Table 1:** Nanosized components of the decontamination formulations.

NPs		Blank (SD) *	S1	S2	S3	S4
	Sample					
ZnO (wt.%)		0	0.1	0.5	1	2
TiO_2_ (wt.%)		0	0.1	0.5	1	2
Zeolite (wt.%)		0	0.1	0.5	1	2

* SD: 2-ethoxyethanol, monoethanolamine, sodium hydroxide, isopropyl alcohol, SDS. S1, S2, S3 and S4, contain, in addition to SD, the corresponding amounts of NPs mentioned above.

## Data Availability

The data presented in this study are available on request from the corresponding author.
